# An early report: a modified porphyrin-linked metronidazole targeting intracellular *Porphyromonas gingivalis* in cultured oral epithelial cells

**DOI:** 10.1038/ijos.2017.31

**Published:** 2017-09-29

**Authors:** Ping Ye, Jiho Chang, Lin Feng Foo, Benjamin C-M Yap

**Affiliations:** 1 Institute of Dental Research, Centre for Oral Health, Westmead Hospital, Westmead, Australia; 2 Affiliation of Faculty of Dentistry, the University of Sydney, Sydney, Australia

**Keywords:** porphyrin-linked metronidazole, *Porphyromonas gingivalis*, periodontitis, oral epithelial cells

## Abstract

*Porphyromonas gingivalis (P. gingivalis)* has a strong association with the pathogenesis of periodontal disease. Recurrence of periodontal disease following therapy is attributed to numerous factors, and of growing interest is the potential problem of intracellular bacteria that are able to persist and multiply within the host cell, thereby facilitating relapse of infection. The effect of antibiotic therapy in controlling *P. gingivalis* is questionable. Accordingly, while metronidazole is very effective against anaerobic extracellular *P. gingivalis* by disrupting the DNA of anaerobic microbial cells, this antibiotic does not effectively penetrate into mammalian cells to inhibit intracellular bacteria. Therefore in the present study, a modified porphyrin-linked metronidazole adducts, developed in our laboratory, was used to kill intracellular *P. gingivalis*. A series of experiments were performed, including cytotoxicity assays and cellular uptake of adducts by flow cytometry coupled with live cell imaging analysis, *P. gingivalis* invasion and elimination assays, and the analysis of colocalization of *P. gingivalis* and porphyrin-linked metronidazole by confocal laser scanning microscopy. Findings indicated that *P. gingivalis* and porphyrin-linked metronidazole were colocalized in the cytoplasm, and this compound was able to kill *P. gingivalis* intracellular with a sufficient culture time. This is a novel antimicrobial approach in the elimination of *P. gingivalis* from the oral cavity.

## Introduction

Periodontitis are communicable diseases spread by bacteria. *Porphyromonas gingivalis (P. gingivalis)* is a keystone pathogen that has strong association with the initiation and progression of periodontitis.^1, 2^ The severity of the disease varies, and if allowed to progress over time can result in inflammatory destruction of alveolar bone and the subsequent loss of teeth. However, periodontitis is a multifactorial disease rather than one caused by a single pathogen, and treatment by physical debridement and irrigation has long been considered an effective means of primary therapy. Recurrence of disease is also attributed to numerous factors, and of growing interest is the possible contribution of intracellular bacteria that are able to persist and multiply within the host cell,^3^ thereby causing relapse of disease processes.^4, 5^

The treatment of periodontitis aims to reduce pathogenic bacteria load on oral tissues with resulting stability of the periodontal attachment apparatus. This can be achieved through surgical means alone or with adjunctive antimicrobial regimes.^6^ Systemic antibiotics have been shown to have benefit in reducing the bacterial load and improving clinical outcomes.^7^ However, the use of broad-spectrum antibiotics carries many side effects, including a potential destruction of beneficial bacteria species and the generation of antibiotic resistance.^8, 9^ A pathogen-specific antimicrobial compound would be ideal in such cases.

Nitroimidazoles such as metronidazole inhibits anaerobic bacteria by its nitro group, which can be reduced by an electron transport protein in anaerobic bacteria.^10, 11^ The reduced metronidazole causes strand damage in the DNA within bacterial cells. Mammalian cells lack the enzymes to reduce the nitro group of these agents, therefore, metronidazole alone does not penetrate into mammalian cells to inhibit intracellular bacteria,^11, 12^ even though metronidazole diffuses well into most tissues and various body fluids.^9^ Systemically administered metronidazole can diffuse into the periodontal tissues and reach deep periodontal pockets through serum to inhibit *P. gingivalis.*^13^ Metronidazole can also be administered locally that has guaranteed the higher therapeutic dose to be delivered inside the pocket, inhibiting extracellular *P. gingivalis*.^13^

*P. gingivalis* exhibits specific features, including frimbriae, extracellular membrane vesicles, polysaccharide capsule, lipopolysaccharide and potent proteases, and the gingipains (RgpA, Kgp),^14^ which allow it to both infiltrate the oral epithelium and localize intracellularly. *P. gingivalis* is unique in that it requires iron and protoporphyrin IX for metabolism but is unable to synthesize the porphyrin ring.^15^ It utilizes gingipains to obtain haem or haem-containing proteins from the environment. This feature represents a specific gateway for addressing the control of this pathogen.

Iron is an essential element for all life. Despite being one of the most abundant elements, it is not readily available as most of the iron within mammalian tissues is sequestered in haem proteins. As a result, microorganisms have evolved special means of extracting iron. Many Gram-negative bacteria produce siderophores, chelating agents that can form soluble Fe^3+^ complexes. *P. gingivalis* does not produce siderophores.^16, 17, 18^ Instead, it acquires iron by taking up the protoporphyrin IX/iron complex (haem) through a specific membrane receptor known as HA2, a component of the gingipains.^19^ Porphyrins are a group of heterocyclic macrocycles composed of four modified pyrrole subunits interconnected at their *α* carbon atoms via methane bridges. Protoporphyrin IX refers to a porphyrin with two propionic, four methyl and two vinyl side chains and it forms haem by taking up an Fe^2+^.^20^ Although *P. gingivalis* relies upon protoporphyrin IX/iron complex for acquisition of iron for survival, the bacterium must also acquire protoporphyrin IX from the environment as it lacks the biosynthetic pathway for the synthesis of the tetrapyrrole ring.^21^

The present study aims to determine the possibility of killing intracellular bacteria using adducts of modified porphyrin linked to metronidazole, an antibiotic developed in our laboratory shown to be very effective against this anaerobic bacterium.^22, 23^ The technique of delivery of metronidazole into the cell is unique, utilizing the ability of *P. gingivalis* to efficiently capture and internalize porphyrins to support basic metabolism. This establishes a potential method for specifically targeting an intracellular pathogen through a specific form of a “Trojan Horse”. The experiment is conducted *in vitro* with the coculture of epithelial cells and *P. gingivalis* and the subsequent introduction of a chemically modified porphyrin-linked metronidazole compound, to confirm the colocalization of *P. gingivalis* and the porphyrin–antibiotic adducts.

## Materials and methods

### Antimicrobial agent

**Figure 1:**
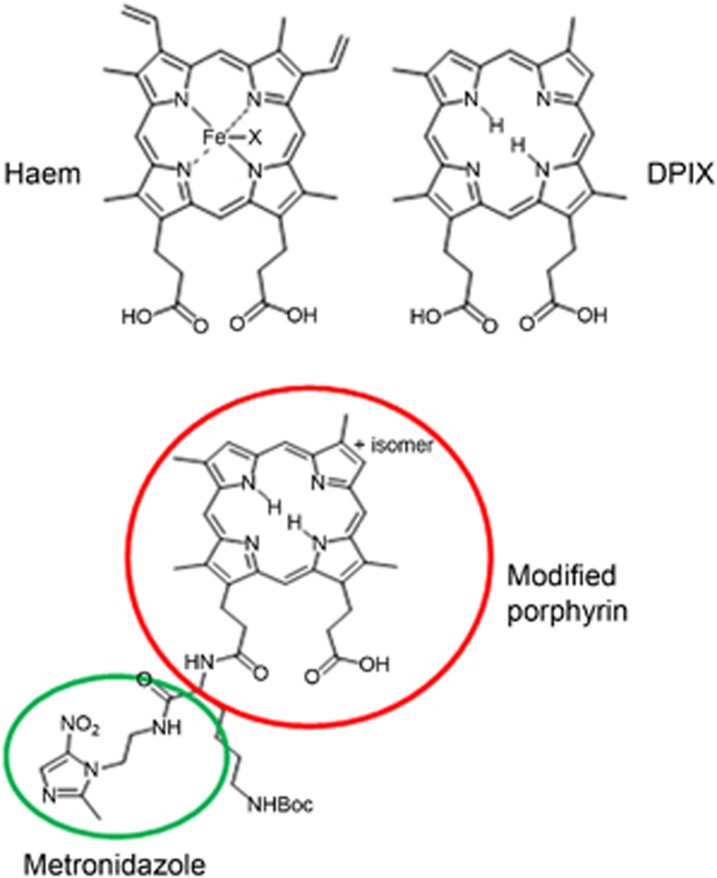
**The structure of a modified porphyrin-linked metronidazole.** DPIX (derivatization of deuteroporphyrin IX) is a modified haem compound without the vinyl groups and iron.

The detailed synthesis of the modified porphyrin-linked metronidazole adducts (Figure 1) from our laboratory has been described previously.^22, 23^ After synthesis, this compound was dissolved in dimethyl sulphoxide (DMSO) to the desired concentration before use. A modified porphyrin not linked to metronidazole was used as a control.

### Oral epithelial cell culture

The epithelial cell line (H413), derived from a human oral squamous cell carcinoma,^24^ displays stratified epithelial cell morphology in culture. H413 cell clonal lines were established using a limit dilution method in our laboratory as described previously.^25^ H413 clone-1 cells exhibiting both characteristic epithelial morphology and high CD24 expression were chosen for this study.^25^ The line was cloned to produce a uniform population suitable for precise experimentation and to select a clone by growth pattern including typical epithelial morphology and for high expression of CD24 to mimic this important feature of the epithelial attachment to the tooth and diseased lining epithelium of periodontitis.^26, 27^ The cloned cells were cultured in Eagle’s Minimum Essential Medium (MEM, Joklik modification, Sigma, Castle Hill, NSW, Australia) and 10% fetal calf serum (FCS, CSL Limited, Parkville, VIC, Australia) at 37 °C in 5% CO_2_. Cultures were harvested with TrypLE Express (replacement for 0.05% trypsin/ethylenediaminetetraacetic acid (EDTA) in phosphate-buffered saline (PBS), Invitrogen, Mulgrave, VIC, Australia) and subcultured every 3 days.

### Lactate dehydrogenase assay

The lactate dehydrogenase (LDH) assay was carried out to rule out the toxic effects of the compound on H413 (clone-1) oral epithelial cells and this was repeated to yield reproducible results. This assay provides a means of measuring either the number of cells via total cytoplasmic LDH or membrane integrity as a function of the amount of cytoplasmic LDH released into the cell medium. A 96-well confluenced cell culture was treated with different concentrations of compounds (20, 40, and 80 μmol·L^−1^, dissolved in 0.1%, 0.2%, and 0.4% DMSO) and DMSO (0.1%, 0.2%, and 0.4%) as controls for up to 7 h. After treatment, cell media were collected at different time points (1–7 h) and 100 μL of each supernatant from each well was transferred to a clean 96-well flat-bottom plate, and enzymatic analysis was carried out. Analysis utilized LDH substrate solution (cat. no.: L2402, Sigma) and LDH dye solution (cat. no.: L2277, Sigma) according to the manufacturer’s procedure. Results were read using an enzyme-linked immunosorbent assay plate reader (Bio-Rad, Gladesville, NSW, Australia) with absorbance at a wavelength of 490 nm, against the background absorbance of 655 nm, and this value was subtracted from the primary wavelength measurement (490 nm). The LDH activities (μU·mL^−1^) of the samples were interpreted from a standard curve for each experiment.

### Uptake of modified porphyrin adducts into cells

Flow cytometry was used to evaluate the cellular uptake of the modified porphyrin-linked metronidazole adducts. Confluenced cell cultures in 12-well plates were treated with different concentrations of adducts (20 and 40 μmol·L^−1^, dissolved in DMSO) for the following time periods, 15 min, 30 min, 1 h, 2 h, and 4 h. Cells were washed in cold PBS and collected by TrypLE Express (Life Technology, Mulgrave, VIC, Australia). After centrifugation, cells were resuspended in cold PBS containing 2% FCS with 0.1% sodium azide in 75 mm tubes and 20 000 cells in 300 μL. Cells were then examined using FACScan and analysed using the CellQuest software (Becton Dickinson, Macquarie Park, NSW, Australia). Porphyrin not linked to metronidazole was provided as a negative control. Each assay was repeated in three separate experiments.

Live cell imaging analysis that determined the penetration/localization of this compound (auto-fluorescence in red) for up to 4 h was described in Supplementary Data.

### Bacterial cell culture

*P. gingivalis* (ATCC 33277 strain) from stock was inoculated into enriched CDC anaerobic broth (Trypticase peptone 10 g, Trypticase soy broth 10 g, Yeast extract 10 g, NaCl 5 g, L-cysteine 0.4 g), supplemented with haemin (5 μg·mL^−1^, Sigma) and menadione (5 μg·mL^−1^, Sigma) and grown in an anaerobic chamber (85% N_2_, 5% CO_2_, and 10% H_2_) for primary culture. Bacterial numbers were estimated by reference to the standard curve determined by absorbance at 600 nm OD=0.8 (1 × 10^9^·mL^−1^) and collected in the late exponential phase. It was considered important to ascertain whether *P. gingivalis* produces cytotoxic effects over the time course of the study, as infected cells could be more sensitive to the adducts and to DMSO. The cytotoxic effects of epithelial cells exposed to *P. gingivalis* has been assessed up to 48 h^28^ (method refers to LDH assay).

### Blood agar assay

The confluent epithelial cells (2 × 10^5^·cm^−2^) were passaged from the flasks into 12-well cell culture plates. Before culture with bacteria, the medium was discarded and the cells were washed three times in PBS and changed with fresh medium MEM (Joklik modified, Sigma) containing 10% FCS without antibiotics. There were six combinations (duplicates) of epithelial cells treated with *P. gingivalis* or antibiotics or porphyrin compound included in the experimental design: (1) Cells with no antibiotics, no *P. gingivalis* and porphyrin not linked to metronidazole were used as controls. (2) Cells were exposed to *P. gingivalis* at a multiplicity of infection of 100 cells per one epithelial cell.^29^ The bacteria were then allowed to invade the cells for 1.5 h^30, 31^ at 37 °C in an atmosphere of 5% CO_2_–95% air. (3) and (4) To confirm intracellular bacterial growth, cells were infected with *P. gingivalis* (1.5 h), following different treatments of metronidazole (20 or 200 μg·mL^−1^, Sigma) and gentamycin (30 or 300 μg·mL^−1^, Sigma) for an additional 1 h incubation, respectively. The desired concentrations of metronidazole (200 μg·mL^−1^ Sigma) and gentamycin (300 μg·mL^−1^ Sigma) were combined for 1 h to kill adherent bacteria from the cell surface (extracellular bacteria).^30, 31^ (5) To confirm that this compound could kill intracellular bacteria, cells were infected with *P. gingivalis* (1.5 h), following the treatment of metronidazole (200 μg·mL^−1^, Sigma) and gentamycin (300 μg·mL^−1^, Sigma) for an additional 1 h incubation, and then were incubated with a modified porphyrin-linked metronidazole at different concentrations (10, 20, 30, and 40 μmol·L^−1^) for up to 4 h. (6) To confirm that this compound could kill both extracellular and intracellular bacteria, cells were infected with *P. gingivalis* (1.5 h), following the treatment of this compound (40 μmol·L^−1^) for up to 6 h without an additional 1 h combined incubation of antibiotics. Up to 6 h incubation with above different treatments of *P. gingivalis* and antibiotics controls were included.

After different treatments, the media were subsequently discarded and the cells were rinsed three times with PBS to remove any residual bacteria/antibiotics or porphyrin adducts. The cells were harvested by scraping and lysed with sterile, distilled water 200 μL for 15 min, then transferred to an anaerobic chamber, and plated onto blood agar plates comprising of bacteriological agar supplemented with haemin (5 μg·mL^−1^, Sigma), menadione (5 μg·mL^−1^, Sigma) and 5% lysed sheep’s blood. The plates were allowed to equilibrate by placing under anaerobic conditions (85% N_2_, 5% CO_2_, and 10% H_2_) for a minimum of 24 h before use. Approximately 90%–95% of cells in each well were harvested through this process. The plates were evaluated after 4 days. All individual cell culture experiments were performed using duplicate wells and each experiment was performed three times.

Meanwhile, disruption of epithelial monolayers by *P. gingivalis* was observed at hourly intervals, for up to 48 h under a phase-contrast microscope (Zeiss D-7082, Oberkochen, Germany) and images were captured by a camera from Nikon (Coolpix 4500, Nikon Inc., Melville, NY, USA).

### Colocalization of *P. gingivalis* and modified porphyrin-linked metronidazole

Confluent H413 clone-1 cells (2 × 10^5^·cm^−2^) grown on eight-well chamber slides (ibidi, Martinsried, Germany) were treated with *P. gingivalis*, or treated with antibiotics or porphyrin-linked metronidazole (as above), washed in PBS, then fixed with 4% paraformaldehyde/PBS for 1 h, permeabilized with 0.1% Triton-X100/PBS for 15 min, and blocked with 3% BSA/PBS for 1 h, and then probed with mouse monoclonal antibody IIB2 specific for gingipains as a primary antibody (5 μg·mL^−1^)^32^ for 1 h at 37 °C. After washing with PBS, fluorochrome-conjugated secondary antibody rabbit anti-mouse IgG Alexa fluor 488 (1:100, Invitrogen) was added for 1 h at 37 °C. As a negative control, the primary antibody was replaced with isotype control antibody IgG (DAKO, North Sydney, NSW, Australia). Slides were washed with PBS and mounted with ProLong Gold antifade reagent with DAPI (Molecular Probes, Invitrogen). Live cells without fixation were to determine the penetration/localization of this compound (auto-fluorescence in red) for up to 4 h. 3D reconstructions were built up by *z*-stack images using the 3D Olympus Fluoview software (Shinjuku-ku, Tokyo, Japan).^33^

Confocal images were captured with an Olympus Fluoview (FV) 1000 system. All fluorescence images prepared with confocal acquisition software (FV10-ASW 4.2) were stored and exported as TIF image files.

### Statistical analysis

All data where necessary were analysed by paired *t*-test (mean±S.D., two-tailed, 95% confidence interval range) from at least three consecutive experiments. *P*<0.05 was considered statistically significant. Image J (National Institutes of Health (NIH), USA) analysis was for bacterial counting and Huygens Professional software (https://svi.nl/HuygensProfessional) was used for analysis of colocalization.

## Results

The optimal concentration of killing bacteria was 40 μmol·L^−1^ for this compound in the oral epithelial cell culture model.

### Measurement of a function of cell membrane integrity

**Figure 2:**
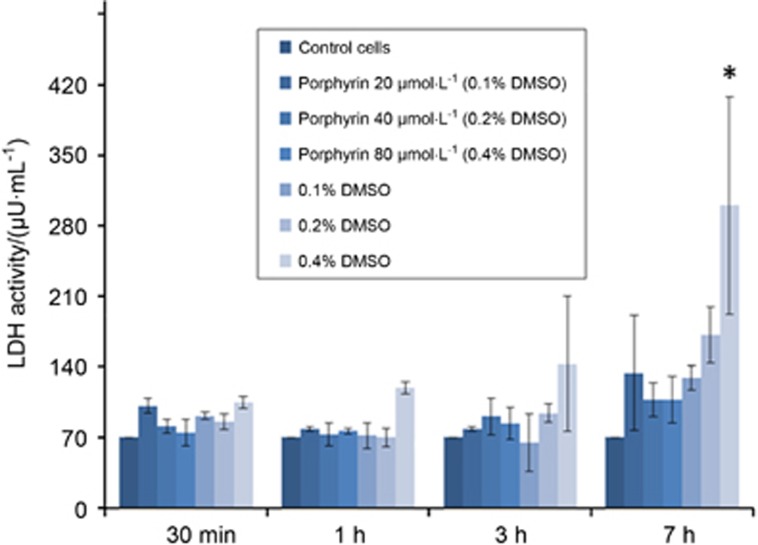
**Measurement of a function of cell membrane integrity.** The LDH assay indicated that the exposure of up to 40 μmol·L^−1^ of porphyrin adducts (dissolved in DMSO) to the cultured oral epithelial cells for up to 7 h had no toxic effect when compared with the control cells. However, the exposure of 80 μmol·L^−1^ adducts of DMSO (0.4%) at 7 h resulted in a significant change when compared with the control cells. 1 Unit (U) is the amount of LDH that catalyses the reaction of 1 μmol of substrate per minute (*P<0.05). DMSO, dimethyl sulphoxide; LDH, lactate dehydrogenase.

It is essential to assess the function of cell membrane integrity while presented with the porphyrin-linked metronidazole compound. The LDH assay indicated that it was safe to expose up to 40 μmol·L^−1^ of modified porphyrin adducts to the cultured oral epithelial cells for up to 7 h compared with the control cells (Figure 2). Assessment of epithelial cells exposed to *P. gingivalis* (ATCC 33277 strain) has no cytotoxic effects up to 24 h (data not shown).

### Cells uptake of modified porphyrin adducts measured by flow cytometry and confocal laser scanning microscopy

**Figure 3:**
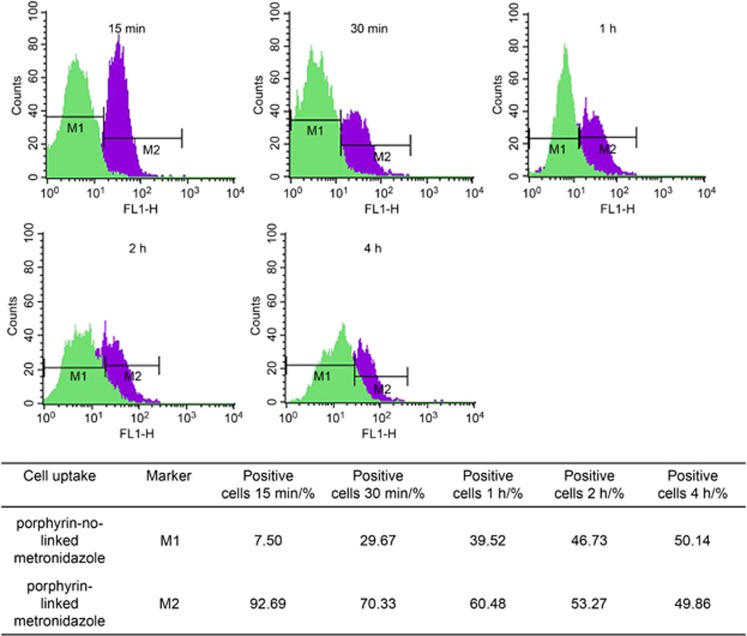
**Determinations of the estimated percentage of positive cells’ uptake of porphyrin adducts.** Control cells with porphyrin not linked to metronidazole (left, in green) were used to set the markers (M1) that defined the negative cell population. This marker was copied to the overlap histogram where the cells with porphyrin-linked metronidazole (right, in purple) were displayed. M2 was then set to indicate the positive cell population. The data showed rapid and major cell uptake of porphyrin-linked metronidazole adducts (40 μmol·L^−1^) starting at 15 min and then reduced positive cells during a time course (up to 4 h).

The porphyrin has a characteristic auto-fluorescence signal (no need of fluorescent label) and is able to penetrate into the cells. Figure 3 showed rapid and major cell uptake of porphyrin-linked metronidazole adducts (40 μmol·L^−1^) at 15 min and then a reduced number of positive cells during a period of time (up to 4 h). The efflux of adduct out of the cells could be degraded. Porphyrin not linked with metronidazole served as a negative control. The flow cytometric analysis monitored both membrane-bound and intracellular porphyrin-linked metronidazole adducts. Live cell imaging analysis determined the penetration/localization of this compound (auto-fluorescence in red) for up to 4 h was shown in Supplementary Data Supplementary Figures S1 and S2.

### Elimination of *P. gingivalis* by modified porphyrin adducts

**Figure 4:**
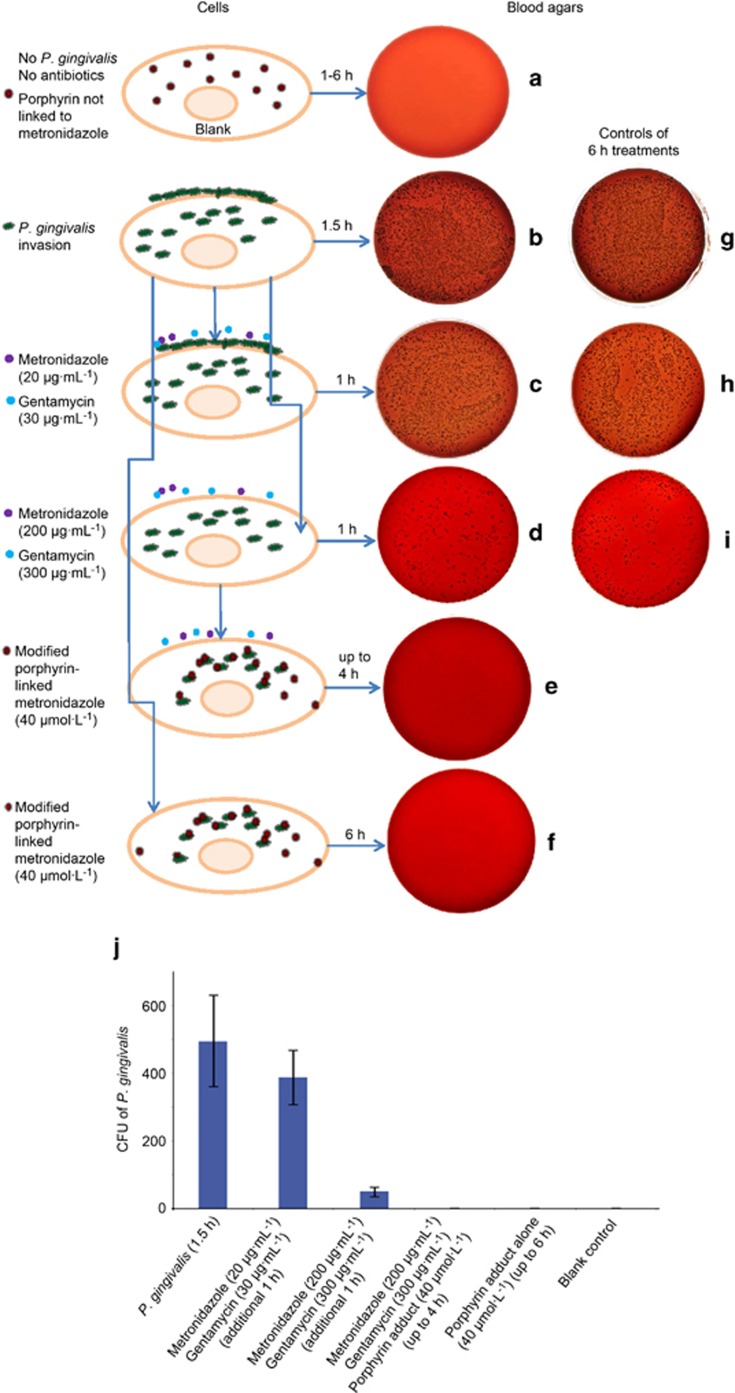
**Blood agar colonies (CFU of *P. gingivalis*) that gave an indication of the efficacy of the porphyrin compound.** (**a**) A negative control blood agar plate indicating no contamination. (**b**) Strong growth from the intracellular and adherent *P.* gingivalis culture for 1.5 h. (**c**, **d**) Invaded *P. gingivalis* treated with metronidazole (20 or 200 μg·mL^−1^) and gentamycin (30 or 300 μg·mL^−1^) for an additional 1 h, respectively, to compare whether there was any differences in killing adherent (extracellular) bacterial cells. Adherent/extracellular (surface-bound) bacteria were only eliminated with a combination of high levels of metronidazole (200 μg·mL^−1^) and gentamycin (300 μg·mL^−1^) (**d**). This represented intracellular infection of *P. gingivalis.* (**e**, **f**) Representative of cells treated with antibiotics and porphyrin compound or porphyrin compound only, for up to 4 and 6 h. All bacteria were eliminated. Panels (**g**–**i**) showed 6 h incubations with different treatments of *P. gingivalis* and antibiotics as controls. Bacteria were similar amounts with early time treatments (**b**–**d**), respectively. (**j**) The summary of the average counting numbers of intracellular *P. gingivalis* colonies (CFU) from blood agar cultures (**a**–**f**) at 4 days. CFU, colony forming units.

*P. gingivalis* was given sufficient time (4 days) to form individual black colonies on blood agar plates. Control cells without antibiotics, *P. gingivalis* and porphyrin not linked to metronidazole had no bacterial growth (Figure 4a) while the wells containing only *P. gingivalis* (1.5 h) demonstrated strong growth of intracellular and adherent bacteria (Figure 4b). A low level of a metronidazole (20 μg·mL^−1^) and gentamycin (30 μg·mL^−1^) combination for an additional 1 h resulted in a similar number of colonies (Figure 4c) similar to the invading by *P. gingivalis* (Figure 4b). When cells were treated with a combination of high levels of metronidazole (200 μg·mL^−1^) and gentamycin (300 μg·mL^−1^), extracellular bacteria were eliminated after an additional 1 h culture, suggesting intracellular infection only (Figure 4d). In our experiments, which included the incubation of the porphyrin compound (10, 20, 30, and 40 μmol·L^−1^) with or without antibiotics for up to 4 and 6 h, there was complete elimination of bacterial growth using a 40 μmol·L^−1^ concentration of this compound (Figure 4e and 4f). This suggests that the compound is able to kill the intracellular bacteria or both intracellular and extracellular bacteria at 40 μmol·L^−1^. For treatments with different concentrations of porphyrin-linked metronidazole (10, 20, and 30 μmol·L^−1^) could reduce microbial load, but there is no completed elimination of *P. gimgivalis* for up to 4 or 6 h (data not shown). Figure 4g–4i showed 6 h incubation with different treatments of *P. gingivalis* and antibiotics controls. Figure 4j displayed the numbers of black colonies formed (colony forming units) to give a clear indication of the efficacy of different treatments from Figure 4a–4f.

Extensively disrupted monolayers for up to 48 h by *P. gingivalis* invasion were observed under a phase-contrast microscope. This suggests that the bacteria were able to survive and spread within the epithelial cells despite not being contained in an anaerobic environment for a limited period of time (data not shown).

### Penetrated modified porphyrin adducts colocalized with *P. gingivalis*

**Figure 5:**
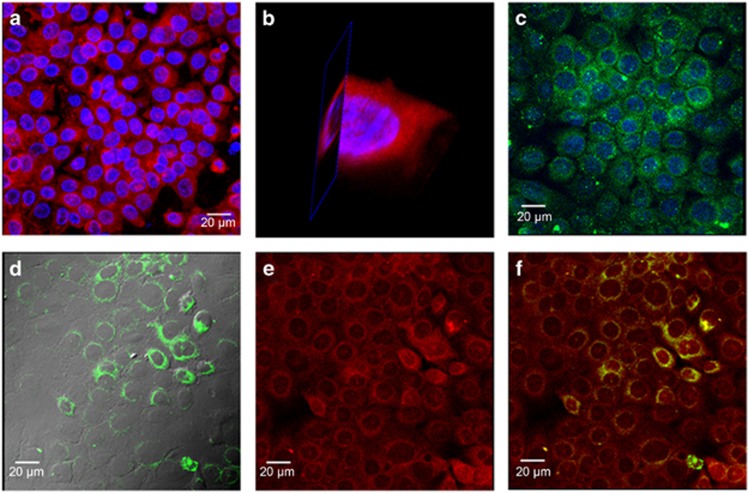
**Live cell imaging of 3D reconstruction and colocalization of porphyrin adducts and pathogen.** (**a**) Live cell imaging of *z*-stack projection at 3 h culture showed porphyrin-linked metronidazole adducts (in red, nuclei in blue) within the cytoplasm of epithelial H413-1 cells by confocal laser scanning microscopy. (**b**) 3D reconstruction with sliced images of one cell confirmed the compound (in red, nuclei in blue) localized in the cytoplasm. (**c**) Adherent and intracellular *P. gingivalis* shown at 1.5 h bacterial invasion to the cells (bacteria in green, nuclei in blue). (**d**) Intracellular *P. gingivalis* showing perinuclear spots in green (grey colour was a differential interference contrast (DIC)) after the killing of extracellular bacteria by combined antibiotics for additional 1 h. (**e**) Demonstration of the porphyrin adducts (in red) colocalized with *P. gingivalis* (in green, **d**) within the cytoplasm around nuclei (perinuclear) of epithelial cells in yellow/orange colour (**f**) at additional 1.5 h culture of this compound.

Using confocal microscopy, it was possible to identify the colocalization of the modified porphyrin adducts and the bacteria. The porphyrin adducts exhibited auto-fluorescence at different wavelengths, but fluorescence was observed to be stronger in the red range (600–650 nm) and was localized in the cytoplasm by analysis of *z*-stack projection images (Figure 5a) and 3D reconstruction images (Figure 5b). Immuno-stained intracellular and adherent *P. gingivalis* at 1.5 h was shown in Figure 5c. Figure 5d showed intracellular *P. gingivalis* following the killing of extracellular bacteria by the combined metronidazole (200 μg·mL^−1^) and gentamycin (300 μg·mL^−1^) treatment for an additional 1 h. Figure 5e showed that the modified drug (in red) was colocalized with intracellular pathogen *P. gingivalis* (in green, Figure 5d) in yellow/orange colour (Figure 5f) within the cytoplasm around the nuclei at 1.5 h incubation.

## Discussion

In this study, strong growth of intracellular and adherent *P. gingivalis* suggests that the bacteria were able to survive within the epithelial cells despite not being contained in an anaerobic environment for a limited period of time. Cellular invasion is an important survival mechanism by which bacteria are able to evade host immunological defences. *P. gingivalis* has been previously shown to use this mechanism for survival.^29, 34, 35^ For example, some studies demonstrated that *P. gingivalis* is able to exit infected cells and reinfect new host cells over time 24 and 48 h.^34, 35, 36^ Therefore, intracellular transmission from cell to cell without host cell lysis and without passing through the extracellular space could be used as an important survival mechanism by this periodontal pathogen.^34, 35^

In the present study, we demonstrated that *P. gingivalis* was able to internalize^36^ in epithelial cells while remaining pathogenic. The capacity of *P. gingivalis* to disrupt epithelial monolayers has been attributed to the proteolytic action of the gingipains such as RgpA^37^ and the capacity of a gingipain null mutant to disrupt multilayered epithelia is markedly reduced.^2^ Disruption of adhesion junctions in epithelia is implicated in the facilitation of invasion by *P. gingivalis* and intracellular infection by this organism.^38^ High molecular weight gingipains also contain a C-terminal adhesin or so-called haemagglutinin domain. Our recent analysis of structurally defined adhesin domain components indicated that the entity termed K2^39^ has structural homology to a class of mammalian intercellular adhesins. K2 also disrupts epithelial barrier function.^33^ Impairment of epithelial barrier function leading to enhanced penetration of microbial products is central to the concept that destructive periodontitis is an immuno-pathological response to microbial products.^27^

The idea of using metronidazole linked with porphyrin, a “Trojan horse” approach as described in the text, is inspiring. High doses of antibiotics seemingly were ineffective in killing the bacteria in virulence (gingipains) location. Porphyrin is required by *P. gingivalis* to survive as it is unable to synthesize the compound. Modification of the porphyrin to contain a specific antimicrobial agent could have affected the *P. gingivalis* in an unpredictable manner or it could have had no effect at all or even promoted growth. Despite this, our experiments indicate that the modified porphyrin-linked metronidazole compound was significantly effective in killing the infection of invading *P. gingivalis*. Two different concentrations of the gentamycin and metronidazole antibiotics were used in order to compare whether there was any differences in killing adherent (extracellular) bacterial cells. The lower concentration revealed no significant differences in the number of growth colonies, similar to infected cells by *P. gingivalis*. Higher concentrations effectively reduced the number of colonies^30, 31^ but did not manage to kill all intracellular bacteria. Findings from our experiments demonstrated that the subsequent incubation of porphyrin adducts for up to 4 h after the introduction of *P. gingivalis* and combined antibiotics resulted in a concentration-dependent reduction of intracellular *P. gingivalis* with complete elimination using a 40 μmol·L^−1^ concentration of adducts. Adducts could be added to toothpaste or mouthwashes or chewing gums to treat and even prevent human periodontitis. Concentration of topically applied adducts could be highly effective at a modest total load.

## Conclusions

In this report, our experiments indicate that the porphyrin-linked metronidazole compound is able to penetrate oral epithelial cells and kill invading *P. gingivalis* with sufficient culture time. The compound alone was not toxic to these cells in the concentration range studied. Accordingly, there is a potential for this compound to eliminate the pathogen from the oral cavity. Subsequent studies of animal models and clinical trials will be conducted in the future.
