# Supplementary material for: An early report: a modified porphyrin-linked metronidazole targeting intracellular Porphyromonas gingivalis in cultured oral epithelial cells
Source: Int J Oral Sci. 2017 Sep 29;9(3):167–73. doi: 10.1038/ijos.2017.31 (PMC5709547; doi:10.1038/ijos.2017.31)
Supplement: Supplementary Information [file ijos201731x1.pdf]

**Supplementary Data**

# An early report: a modified porphyrin linked metronidazole targeting intracellular *Porphyromonas gingivalis* in cultured oral epithelial cells

Ping Ye[1,2]*, Jiho Chang[1], Lin Feng Foo[1], and Benjamin C-M Yap[1]

[1] Institute of Dental Research, Centre for Oral Health, Westmead Hospital, Westmead, Australia

[2] Affiliation of Faculty of Dentistry, the University of Sydney, Sydney, Australia

## MATERIALS AND METHODS

Live cell imaging analysis determined the penetration/localization of this compound (auto-fluorescence in red) for up to 4 hours. Briefly, Confluent H413 clone-1 cells ($2 \times 10^5/cm^2$) grown on 8-well chamber slides (ibidi, Germany) were treated with modified porphyrin-linked metronidazole (20 and 40 µM, dissolved in DMSO) or DMSO (0.2%) observed by Olympus Fluoview (FV) 1000 confocal system. 3D reconstructions were built up by z-stack images using 3D Olympus Fluoview software.[1]

## RESULTS

Figure S1 showed this compound was being uptake at 15 min, major located at cell surface, and then penetrating into the cells around 35 min, getting stronger expression around 1-3 h, then reducing its expression at 4 h. There was no impact of the porphyrin adducts/DMSO on cell morphology change for up to 4 h. The porphyrin adducts exhibited auto-fluorescence at different wavelengths, but fluorescence was observed to be stronger in the red range (600-650 nm) and was localized in the cytoplasm by analysis of z-stack projection images (Figure S2a) and 3D reconstruction images (Figure S2b).

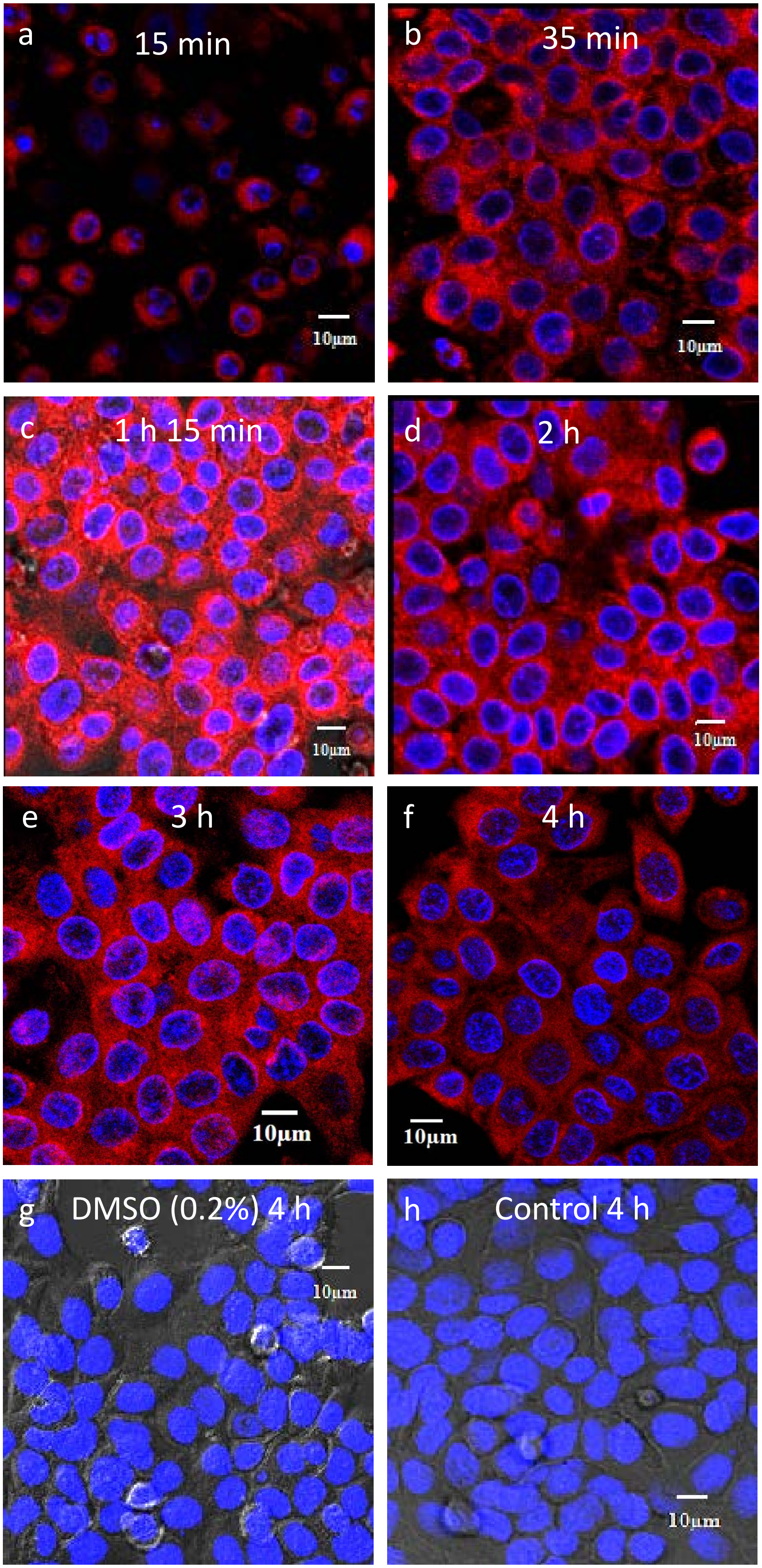
a
15 min
10µm
b
35 min
10µm
c
1 h 15 min
10µm
d
2 h
10µm
e
3 h
10µm
f
4 h
10µm
g
DMSO (0.2%) 4 h
10µm
h
Control 4 h
10µm

**Figure S1** Live cell imaging showed that this compound was uptake at 15 min, major located at cell surface (**a**), and then penetrating into the cells around 35 min (**b**). The compound displayed a strong expression at 1-3 h (**c-e**), and a reduced expression starting at 4 h (**f**). There was no impact of the porphyrin adducts/DMSO on cell morphology change for the experiments (**g**: 0.2% DMSO; **h**: control cells).

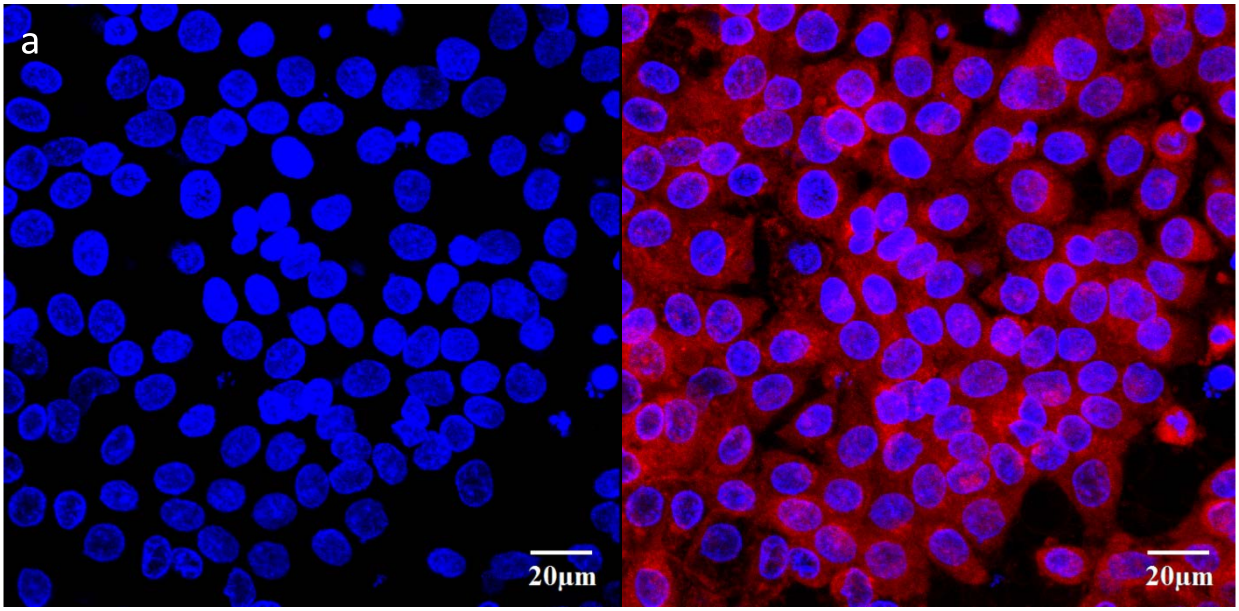


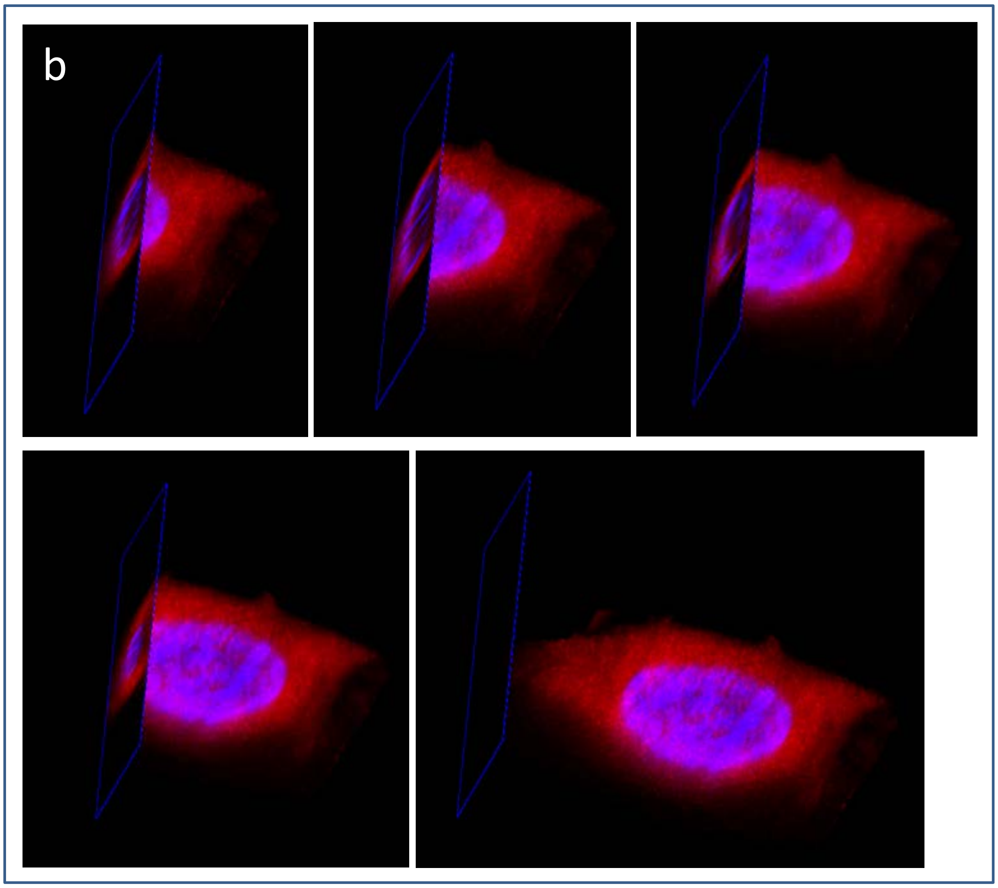

**Figure S2** (**a**) Live cell imaging of z-stack projection at 3 h culture showed porphyrin-linked metronidazole adducts (in red, nuclei in blue) within the cytoplasm of epithelial H413-1 cells by confocal laser scanning microscopy. (**b**) 3D reconstruction with a series of sliced images of one single cell confirmed the compound (in red, nuclei in blue) localised in the cytoplasm.

1. Ye P, Yu H, Houshmandi M. Three/four-dimensional (3D/4D) microscopic imaging and processing in clinical dental research. *BMC Oral Health* 2016; **16**(1): 84.
